# Development of an emergency general surgery process improvement program

**DOI:** 10.1186/s13037-018-0167-z

**Published:** 2018-06-20

**Authors:** Matthew J. Bradley, Angela T. Kindvall, Ashley E. Humphries, Elliot M. Jessie, John S. Oh, Debra M. Malone, Jeffrey A. Bailey, Philip W. Perdue, Eric A. Elster, Carlos J. Rodriguez

**Affiliations:** 1 Department of Surgery, Walter Reed National Military Medical Center, 8901 Rockville Pike, Bethesda, MD 20889 USA; 2 Department of Surgery, Uniformed University of the Health Sciences, 4301 Jones Bridge Road, Bethesda, MD 20814 USA

## Abstract

**Background:**

The Joint Trauma System has demonstrated improved outcomes through coordinated research and process improvement programs. With fewer combat trauma patients, our military American College of Surgeons level 2 trauma center’s ability to maintain a strong trauma Process Improvement (PI) program has become difficult. As emergency general surgery (EGS) patients are similar to trauma patients, our Trauma and Acute Care Surgery (TACS) service developed an EGS PI program analogous to what is done in trauma. We describe the implementation of our novel EGS PI program and its effect on institutional PI proficiency.

**Methods:**

An EGS registry was developed in 2013. Inclusion criteria were based on AAST published literature. In 2015, EGS registrar and PI coordinator positions were developed and filled with existing trauma staff. A formal EGS PI program began January 1, 2016. Pre- and post-program data was compared to determine the effect including EGS PI events had on increasing yield into our trauma PI program.

**Results:**

In 2016, TACS saw 1001 EGS consults. Four hundred forty-four met criteria for registry inclusion. Eighty-two patients had 131 PI events; re-admission within 30 days, unplanned therapeutic intervention, and unplanned ICU admission were the most common events. Capture of EGS PI events yielded a 49% increase compared with 2015.

**Conclusion:**

Overall patient volume and PI events post EGS PI program initiation exceeded those prior to implementation. These data suggest that extending trauma PI principles to EGS may be beneficial in maintaining inter-war military and/or lower volume trauma center readiness.

## Background

In 2013, our military hospital’s trauma service became the primary team for all emergency general surgery (EGS) consultations and admissions. The merger of trauma and acute care patients into one team provided a continuous flow of critically ill patients with acute and complex surgical needs and a cohort of patients that mirrored each other in both surgical acuity and attendant complications.

It was quickly recognized that EGS patients in the combined program presented additional Process Improvement (PI) opportunities as care provided to EGS patients was often not scrutinized to the same extent as our trauma patients, despite sharing hospital rooms and being cared for by the same team of surgeons. Moreover, as American College of Surgeons Committee on Trauma (ACSCOT) verified trauma centers with established PI programs have demonstrated improved outcomes [1], it was anticipated that applying these principles with EGS patients would result in similar improved outcomes.

Additionally, it was acknowledged that expansion of trauma PI to EGS patients, could provide an opportunity to maintain our overall system proficiency at providing critical analysis of the care provided to our patients through the maintenance of its backbone- a strong process improvement (PI) program [2]. As pointed out in National Academies of Science Engineering Medicine report, *A National Trauma Care System: Integrating Military and Civilian Trauma Systems to Achieve Zero Preventable Deaths After Injury*, maximum benefit from PI requires its implementation not only at the provider and facility level but at the systems level as well [3]. Thus, the goal with the combined program was to maintain system proficiency and to provide a program of quality and process improvement for our surgical patients. We hypothesized that through the combined PI program additional opportunities for process improvement would be uncovered and system proficiency would be maintained. The following is a description of the novel method of PI implementation and our approach at maintaining system proficiency.

## Methods

Prior to the September 2011 completion of the Base Realignment and Closure (BRAC) creating the Walter Reed National Military Medical Center (WRNMMC), there were two major military treatment facilities in the National Capital Region (NCR)- Walter Reed Army Medical Center, Washington, DC and the National Naval Medical Center (NNMC), Bethesda, MD. Each institution had its own trauma program and PI strategies. The formal WRNMMC trauma PI began in late 2011 and matured in the following years. At the time of the merger, trauma abstraction and PI focused solely on combat casualties. However, in mid-2012 due to decreasing combat trauma numbers, trauma service data abstraction expanded to include *all* trauma patients admitted to WRNMMC. In 2013, the Trauma and Acute Care Surgery (TACS) service was formally established.

While an informal patient tracking system began when the TACS service was established, in 2015 as part of our evolving TACS PI process, a formal emergency general surgery (EGS) registry was purchased, ACS Collector ™ (2010, Digital Innovation, Incorporated, Forest Hill, MD), to track patients and outcomes. However, EGS encompasses a broad range of both operative and non-operative acute surgical practice. Therefore, to more specifically focus our registry’s scope, we utilized the American Association for the Surgery in Trauma’s (AAST) previously defined set of EGS diagnoses as the basis for our inclusion criteria, (Table 1) [4].

**Table 1:** Inclusion criteria for emergency general surgery patients admitted to the TACS service

Common Clinical Diagnoses	
Gastrointestinal: Abdominal compartment syndrome, abdominal pain, anorectal abscess and fistula, appendicitis, cholecystitis, choledocholithiasis, colitis, diverticulitis, gastrointestinal bleed, hemoperitoneum (non-traumatic), hemorrhoids, intestinal obstructions, intraabdominal and retroperitoneal abscesses, liver abscess, mesenteric ischemia, pancreatitis, peritonitis, ulcer disease.	
Incarcerated or strangulated Hernias: Femoral, incisional, inguinal, paraesophageal and diaphragmatic umbilical, ventral.	
Skin and soft tissue: Abscess, cellulitis, compartment syndrome, fasciitis, necrotizing soft tissue infections, pressure ulcers.	
Other: Sepsis, shock, surgical airway	

For all patients meeting criteria, their electronic medical record (EMR) was abstracted for the first 30 days of hospitalization. Additionally:

1. Surgical intervention was not a requirement for inclusion in the registry provided patients were medically managed by the TACS service during their inpatient stay.
2. Any inpatient consult received by the TACS service that required subsequent follow-up, regardless of whether a surgical procedure was performed, was also included in the registry and their EMR was abstracted for the length of the TACS service consultation.
3. Intraoperative events requiring TACS service intervention, such as repairing an iatrogenic bowel injury, were also included in the registry.

The EGS Registry and PI program were staffed with two personnel from the trauma program- a registrar and a nurse coordinator. The functions of these new EGS positions were created identically to those described in the American College of Surgeons Committee on Trauma “Orange Book” for trauma registrars and Trauma Nurse Coordinators [2]. The EGS PI nurse coordinator makes daily rounds with the TACS team, attends morning report where all surgical consults and admissions from the previous 24 h are discussed, and attends the department’s weekly surgery morbidity and mortality conference when TACS patients are being discussed.

Additionally, on a daily basis, the PI coordinator also reviews inpatient charts EGS patient charts looking for deviations from standard of care, complications, and untoward events or outcomes. To guide chart review, we developed a list of common PI events based upon our existing trauma PI program and guidance from the American College of Surgeons’ National Surgical Quality Improvement Program Operations Manual, 2012, Chicago: American College of Surgeons (Table 2).

**Table 2:** Emergency general surgery process improvement events

Filter	Filter
Superficial SSI	Non-therapeutic Ex-lap
Deep SSI	Transfusions
Organ Space	Graft/Prosthetic/Flap Failure
Wound Disruption	DVT/Thrombophlebitis
Pneumonia	Sepsis
Unplanned Intubation	Septic Shock
Pulmonary Embolus	Re-admit with/in 30 days
Ventilator > 48 h	Anastomotic Leak
Renal Failure (Progressive)	Death
Acute Renal Failure	Pressure Ulcer
UTI	Blood Bank / Lab Issues
CVA/Stroke	Radiology Issues
Coma > 48 h	Unplanned ICU admit
Peripheral Nerve Injury	Unplanned Intervention
RRT	Returned to OR
Cardiac Arrest	Documentation
Myocardial Infarction	Delay in Diagnosis
Incidental Findings	Interfacility Event
Patient Safety	Missed Diagnosis
Positive Cultures	

Much like trauma PI, all EGS PI events undergo primary, secondary, and tertiary levels of review and are presented at weekly PI conferences for discussion and loop closure. Abstraction into the EGS Registry officially began, January 1, 2016. As EGS and trauma patients often utilize similar medical and surgical hospital resources, we compared work volume pre- and post- initiation of the EGS registry / PI program as a surrogate to measuring maintenance of system proficiency. Particular attention was paid to the number of PI events discovered in the pre- and post- periods.

## Results

**Fig. 1:**
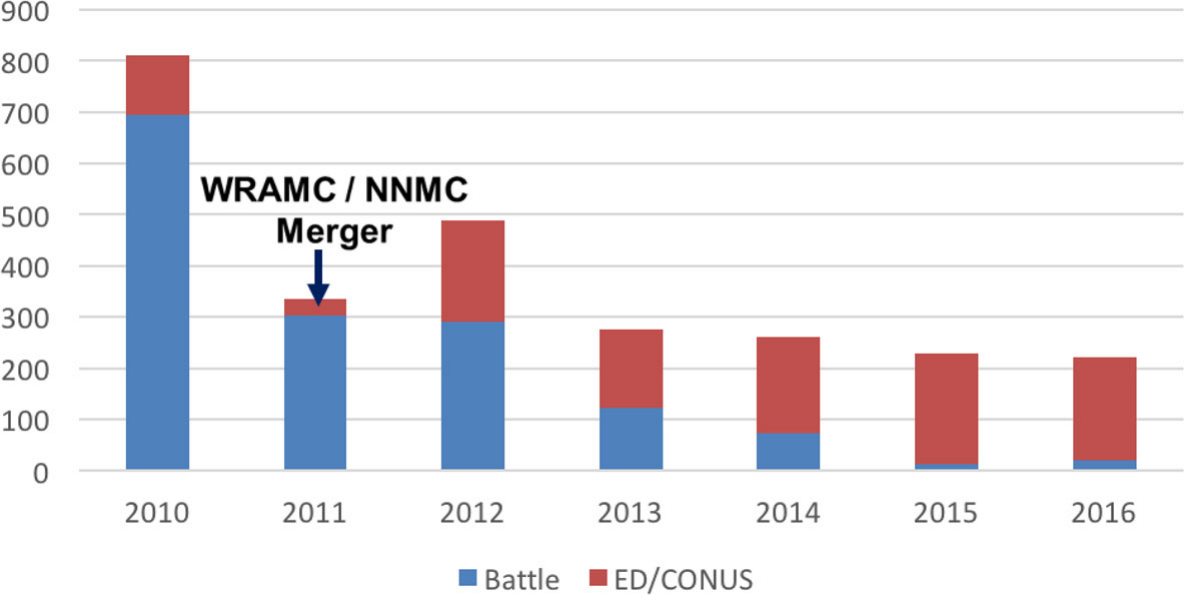
Trauma Admissions: Trend of trauma patient admissions and distribution of Battle versus Non-Battle Injuries since 2010

**Fig. 2:**
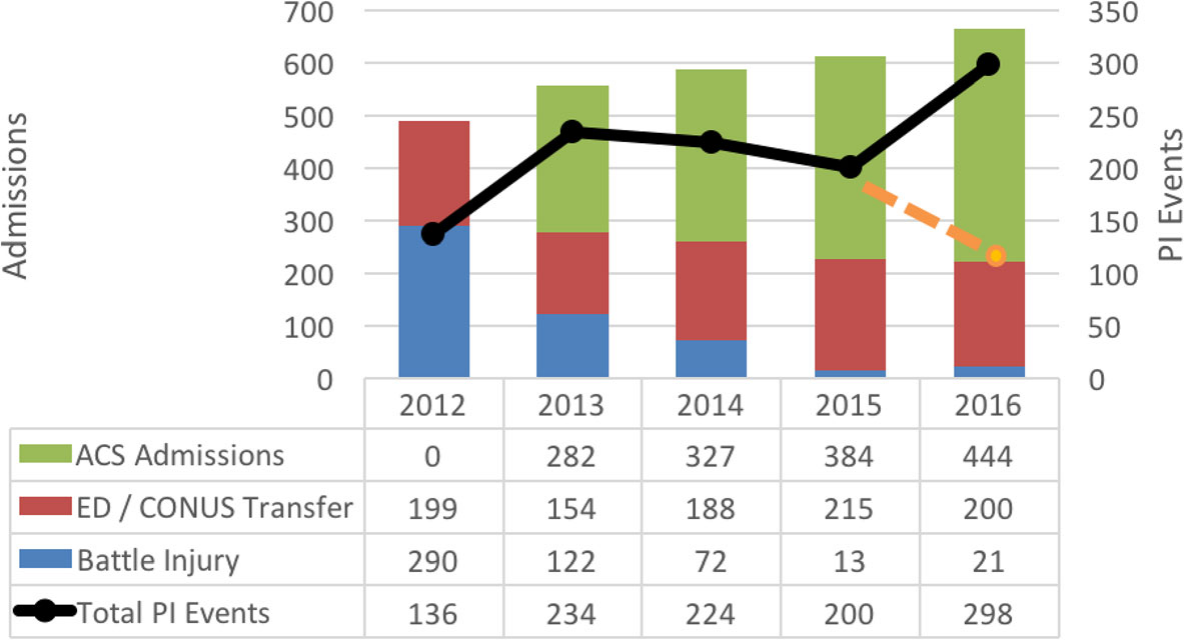
Admissions and PI events over time: Comparison of yearly trauma versus trauma plus acute care surgery patient admissions and trends of PI events. Orange line indicates PI volume contributed by trauma patients in 2016. (PI- Process Improvement)

In 2010, NCR trauma admissions, from oversea casualties, exceeded 800. In 2011 and 2012 those admissions declined to approximately 1/3 and 1/2, respectively, of 2010’s numbers (Fig. 1). The make-up of these casualties changed over time with a larger proportion of patients consisting of non-battle related injuries (NBI). The 2011 decline occurred due to merger logistics between WRAMC and NNMC- where it was necessary to divert some patients to other facilities within the Continental United States Continuum of Care. As merger logistics dissipated, the decline over 2012 was secondary to changes in combat operational tempo and patient regulation back to the US. In the ensuing years, a larger percentage of patients on the TACS team were EGS patients. With the addition of these patients, TACS admissions have increased since 2013 approaching the trauma-only census of 2010. (Fig. 2). In 2016, there was a bimodal distribution for admission trauma age with over half (52%) of the patients admitted distributed in either the 18–29 (25%) or 80–99 (27%) years old age range. Conversely, the acute care, non-trauma patients were distributed in a more normal pattern with the majority of patients (27%) falling in the 50–64 age range.

Trauma PI events decreased from 2012 to 2015 (Fig. 2). However, with the inclusion of EGS PI events, total PI events increased in 2016. If EGS events were not captured, total PI events would have continued to decline into 2016 (dotted line in Fig. 2).

Of the 221 trauma patients in 2016, 104 (47%) had 161 PI events. Seventy-eight of the 161 (48%) events were “admit to non-surgical service.” None of the 78 admissions to non-surgical services were combat casualties. All 78 went through our PI process, had minor injuries (ISS < 9), and were found to have been appropriately admitted- within standards of care. Of the 444 EGS patients abstracted in 2016, there were a total of 82 patients (18%) with 131 PI events (Table 2).

**Fig. 3:**
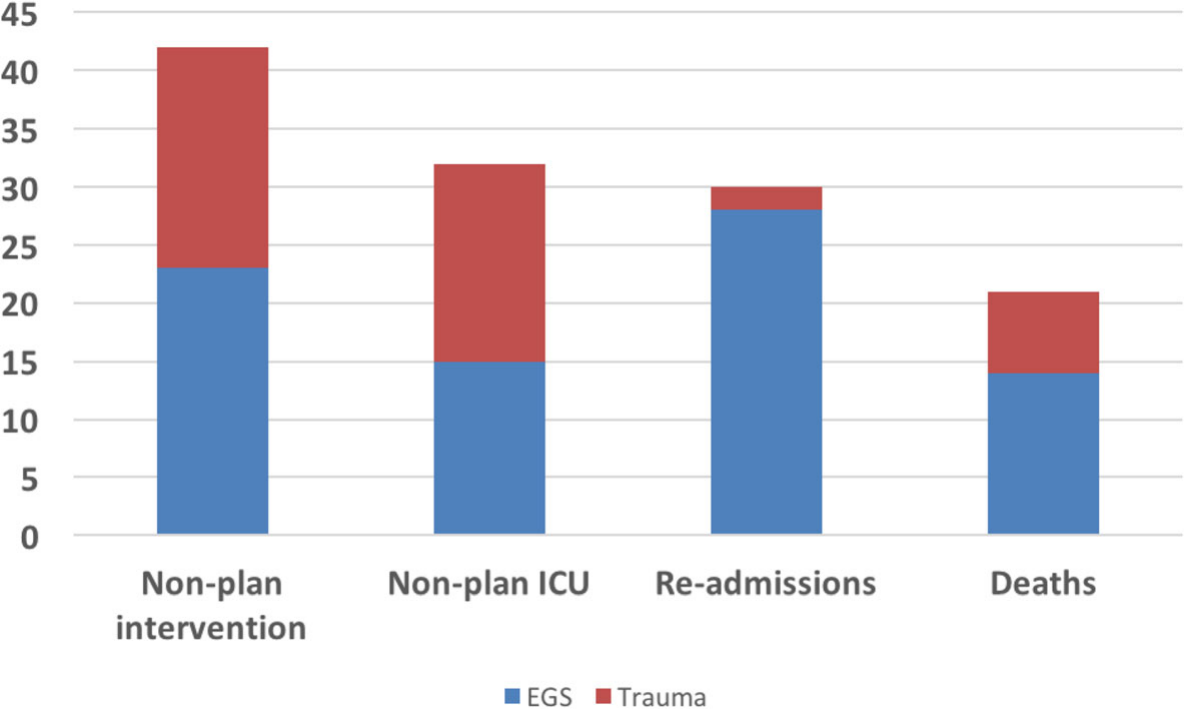
ACS and trauma PI events in 2016: 665 total trauma patients and 298 total PI events. Top four combined PI events are displayed. (ACS- Acute Care Surgery, PI- Process Improvement)

The most common EGS PI event was “re-admission within 30 days” followed by unplanned procedures. Of all the readmission events, 93% were EGS patients versus 7% trauma patients. In addition, EGS accounted for 55% of unplanned procedures. Overall, the inclusion of EGS PI events in 2016 yielded a 49% increase over 2015. The frequency and type of Trauma and EGS PI events for 2016 are shown in Fig. 3.

## Discussion

To our knowledge, we are the first treatment facility to establish an EGS PI program. While other institutions have developed registries and PI programs, no formal program has been published to date [5]. As WRNMMC does not formally participate in the state or regional civilian trauma system or routinely care for civilian trauma patients, our ability to maintain a strong trauma PI program is predicated on transfers and our participation in the Joint Trauma System’s Continuum of Care; therefore, we developed this program, in part, to sustain the proficiency of our process improvement program of our military trauma center. Through our combined TACS team we augmented our military surgical practice, enhanced our established PI program, and brought requisite quality and process improvements to EGS patients.

Moving forward, by investigating our emergency surgery PI events we can identify key areas where our system can improve and/or adapt in order to decrease morbidity and mortality. This is especially important as the emergency surgery patient has a much higher morbidity and mortality compared to non-emergency, elective general surgery patients with respect to infection, multiple organ derangement, and medical error [6–9]. In particular, readmission was most common and relatively new PI event. Further analysis of EGS readmissions will provide more details and identify areas our system can improve upon to limit readmissions in the future.

With the National Academies of Sciences, Engineering, and Medicine committee’s goal of achieving zero preventable deaths after injury, the requirement to have a mature PI program couldn’t be more immediate. To that end, through establishment of our new PI program we have taken action to ensure that we do not become complacent so that we can continue to improve upon the care provided to our patients. It was first recognized almost 40 years ago that preventable deaths could be reduced through an organized trauma system [10]. Since then, several studies have confirmed that the implementation of a trauma system resulted in improved care and decreased mortality [11–14]. As trauma programs matured, facilities began to initiate audit processes to evaluate patient outcomes [13, 15, 16]. Subsequently, these audits formed the basis of more comprehensive trauma process improvement programs. These programs have matured to the point where they include formal trauma registrars, ongoing data collection and prospective analysis, and committees to implement plans and protocols to enhance patient outcomes [15, 17, 18].

While the benefits of a mature trauma system and PI program are well established, EGS programs are nascent and thus there is a paucity of literature on the advantages of a dedicated EGS PI program. While some authors have demonstrated lower morbidity and mortality through a regional, mature EGS system, others have been unable to reproduce these findings attributing the results to their naïve system [19, 20]. However, EGS patients are over twice as likely to have a major complication compared to patients undergoing elective procedures with emergency colorectal surgery having a particularly high complication rate of greater than 50% [6, 7, 21, 22]. Additionally, mortality is also significantly higher as EGS patients are reportedly five times more likely to die as compared to elective surgical patients [6, 7, 21]. The high morbidity and mortality of ACS patients underscores the need for continued efforts to develop mature systems [6].

Since EGS patients share similar resources and processes as trauma patients, merging EGS with a trauma program to create an integrated TACS service makes good sense for both patient and surgical team [23]. Data has shown that combining these two patient populations is feasible and increases the trauma surgeon and trauma team operative experience while also sustaining optimum care of the sick and injured patient [24]. Despite these findings, the ideal EGS model has not been defined and the broad adoption of EGS programs with the inclusion of both EGS and trauma patients under one system has varied [25, 26]. However, as these EGS systems become more widely implemented, institutionalized, and standardized patient outcomes will continue to improve [20, 26]. In addition, although intuitive, the measured contribution of complex acute surgical care to trauma team proficiency has yet to be fully defined or validated.

## Conclusion

We developed a trauma and acute care surgery (TACS) program merging EGS and trauma patients into one service to augment our decline in trauma patient volume. We believe that this will help us maintain a trauma program and system proficiency and enhance our trauma center’s PI program. The integration of EGS into our trauma program has increased patient volume and, therefore, the number of subsequent PI events exceeded our TACS numbers prior to the merger of these patient populations. Extending trauma PI principles to EGS may be beneficial in maintaining trauma center and trauma team readiness. Analysis of our EGS data will provide an ongoing opportunity for exercise and maturation of our military trauma center performance improvement program independent of combat operational tempo.

## Abbreviations

AAST: American Association for the Surgery in Trauma
ACSCOT: American College of Surgeons Committee on Trauma
BRAC: Base Realignment and Closure
EGS: Emergency General Surgery
EMR: Electronic medical record
NBI: Non-battle related injuries
NCR: National Capital Region
NNMC: National Naval Medical Center
PI: Process improvement
TACS: Trauma and Acute Care Surgery
WRNMMC: Walter Reed National Military Medical Center
